# Understanding Neovascularization in Glioblastoma: Insights from the Current Literature

**DOI:** 10.3390/ijms26062763

**Published:** 2025-03-19

**Authors:** Mariagiovanna Ballato, Emanuela Germanà, Gabriele Ricciardi, Walter Giuseppe Giordano, Pietro Tralongo, Mariachiara Buccarelli, Giorgia Castellani, Lucia Ricci-Vitiani, Quintino Giorgio D’Alessandris, Giuseppe Giuffrè, Cristina Pizzimenti, Vincenzo Fiorentino, Valeria Zuccalà, Antonio Ieni, Maria Caffo, Guido Fadda, Maurizio Martini

**Affiliations:** 1 Department of Biomedical, Dental, Morphological and Functional Imaging Sciences, University of Messina, 98125 Messina, Italy; mariagiovannaballato96@gmail.com (M.B.); emanuelagermana@hotmail.it (E.G.); gricciardi1998@gmail.com (G.R.); waltergiordano1997g@gmail.com (W.G.G.); pietrotralongo@gmail.com (P.T.); 2 Istituto Clinico Polispecialistico C.O.T. Cure Ortopediche Traumatologiche s.pa., 98124 Messina, Italy; 3 Department of Oncology and Molecular Medicine, Istituto Superiore di Sanità, 00161 Rome, Italy; mariachiara.buccarelli@iss.it (M.B.); giorgia.castellani@iss.it (G.C.); lriccivitiani@yahoo.it (L.R.-V.); 4 Department of Neurosurgery, Fondazione Policlinico Universitario A. Gemelli IRCCS, 00168 Rome, Italy; quintinogiorgio.dalessandris@policlinicogemelli.it; 5 Department of Human Pathology in Adult and Developmental Age “Gaetano Barresi”, University of Messina, 98125 Messina, Italy; giuseppe.giuffre@unime.it (G.G.); vincenzo.fiorentino@unime.it (V.F.); valeria.zuccala@unime.it (V.Z.); antonio.ieni@unime.it (A.I.); guido.fadda@unime.it (G.F.); 6 Pathology Unit, Papardo Hospital, 98158 Messina, Italy; cristinapizzimenti86@gmail.com; 7 Biomedical and Dental Sciences and Morphofunctional Imaging, Unit of Neurosurgery, University of Messina, 98122 Messina, Italy; mariella.caffo@unime.it

**Keywords:** glioblastoma (GBM), aberrant angiogenesis, strategies of neovascularization, pro-angiogenic factors, emerging therapies

## Abstract

Glioblastomas (GBMs), among the most aggressive and resilient brain tumors, characteristically exhibit high angiogenic potential, leading to the formation of a dense yet aberrant vasculature, both morphologically and functionally. With these premises, numerous expectations were initially placed on anti-angiogenic therapies, soon dashed by their limited efficacy in concretely improving patient outcomes. Neovascularization in GBM soon emerged as a complex, dynamic, and heterogeneous process, hard to manage with the classical standard of care. Growing evidence has revealed the existence of numerous non-canonical strategies of angiogenesis, variously exploited by GBM to meet its ever-increasing metabolic demand and differently involved in tumor progression, recurrence, and escape from treatments. In this review, we provide an accurate description of each neovascularization mode encountered in GBM tumors to date, highlighting the molecular players and signaling cascades primarily involved. We also detail the key architectural and functional aspects characteristic of the GBM vascular compartment because of an intricate crosstalk between the different angiogenic networks. Additionally, we explore the repertoire of emerging therapies against GBM that are currently under study, concluding with a question: faced with such a challenging scenario, could combined therapies, tailored to the patient’s genetic signatures, represent an effective game changer?

## 1. Introduction

Glial tumors, also known as gliomas, are malignant neoplasms that grow in the brain or, less frequently, in the spinal cord, whose cellular origin remains a matter of debate. This kind of tumor presumably arises from improperly reactivated glial cells or from aberrant neural stem/glial progenitor cells, but the different hypotheses are not necessarily mutually exclusive; distinct subtypes might have distinct tumor-initiating cells [1,2,3,4,5,6]. Gliomas can occur at any age, even in children, representing the most prevalent and aggressive primary tumors of the central nervous system (CNS) [7]. The disease is more frequently diagnosed in Caucasians, ranging from 45 to 65 years old [8,9,10,11,12,13]. Epidemiological evidence has also revealed higher incidence and shorter lifespan in males than in females, suggesting the possible existence of sex-related aspects in tumorigenesis, survival rate, and response to therapies [8,12,14,15,16,17]. However, regardless of gender, advanced age appears to be a negative prognostic factor, with poorer outcomes [18,19].

Gliomas are mostly sporadic tumors, although a small fraction of cases (about 5%) show familial features with the occurrence of the disease in two or more members of a family, generally with early onset [20,21]. In this context, recent genome-wide association studies (GWASs) through the identification of 25 putative risk loci (like *TERT* 5p15.33, *MDM4* 1q32.1, *TP53* 17p13.1, *EGFR* 7p11.2, and others) seem to support the scenario of a possible polygenic susceptibility to gliomas. The underlying molecular mechanisms, however, are still poorly understood [21,22,23]. In addition, approximately 1–2% of glial tumors are strictly associated with hereditary diseases, such as neurofibromatosis type 1, Lynch, Li–Fraumeni, Turcot, and Cowden syndromes [17,24]. Furthermore, several potential predisposing factors, including exposure to environmental agents, lifestyle choices, and unhealthy behaviors that may be related to a higher incidence of gliomas, are currently being studied. Individuals with a history of allergies or asthma, curiously, seem less inclined to develop glial tumors. However, to date, exposure to ionizing radiation is the only proven and well-documented environmental risk factor [17,21].

Given the level of heterogeneity, an integrated and layered diagnosis, although challenging, is required to ensure the most appropriate patient management. Specifically, gliomas form a wide collection of low- to high-grade tumors classified in compliance with the 2021 fifth edition World Health Organization (WHO) classification of tumors of the CNS [25,26,27]. Based on histopathological aspects, immunohistochemical features, and molecular/cytogenetic profiles, indeed, adult-type diffuse gliomas are currently categorized into three tumor types, as follows: (1) *isocitrate dehydrogenase* (*IDH*)-mutant and 1p/19q-codeleted oligodendrogliomas, (2) *IDH*-mutant astrocytomas, and (3) *IDH*-wildtype glioblastomas [28]. Furthermore, tumor grading, from grade 1 (the least aggressive) to grade 4 (the most aggressive) in terms of malignancy, is established not only by evaluating different histological criteria (mitosis, nuclear atypia, microvascular proliferation, and necrosis) but also by assessing molecular characteristics [29,30]. Moreover, under the updated classification, the presence of *CDKN2A/B* homozygous deletion is sufficient to consider an astrocytoma IDH-mutant, WHO grade 4, even without microvascular proliferation and/or necrosis. In the same way, an IDH-wildtype astrocytoma falls under the group of glioblastomas in the presence of at least one of the following characteristics: necrosis and/or microvascular proliferation and/or *TERT* promoter mutation and/or *EGFR* gene amplification and/or concomitant gain of chromosome 7 and loss of chromosome 10 [28].

Glioblastoma multiforme (GBM), with a median overall survival (OS) of around 15 months, represents, among gliomas, the most malignant and poorest prognosis form [31,32,33]. Hallmarks such as rapid growth, a highly infiltrative nature, a pronounced tendency to recur, and refractoriness to therapeutic options make GBM essentially incurable [34,35]. A “one-size-fits-all” approach (Stupp protocol) is currently used for glioblastoma patients. Specifically, the standard of care involves an aggressive, multimodal, tripartite strategy: safe surgical debulking, radiotherapy, and adjuvant chemotherapy with the alkylating drug temozolomide (TMZ) [35,36,37]. However, despite ongoing efforts to counteract the inexorable progression of the disease, treatment plan effectiveness is insufficient, leading to only slight improvements in survival rates [38].

One of the key features of GBMs is their extensive vascularization [39]. This signature trait has led to hopes for the success of anti-angiogenic therapies that have emerged in the last decades as a promising treatment option for several types of solid tumors [40]. By starving GBM, indeed, given the great vascular density, it was expected that this could stop or slow down its progression, leading to better prognoses [41]. Furthermore, by normalizing the tumor vascular network, anti-angiogenic therapies would have improved the tissue distribution and effectiveness of chemotherapeutic agents [42]. With these premises, GBM angiogenesis has become an important research focus, and in 2009, the FDA approved bevacizumab monotherapy—a recombinant humanized antibody against VEGF—for recurrent glioblastoma treatment [43,44,45]. However, as suggested by various clinical studies, while angiogenesis inhibitors (usually combined with conventional therapies) tend to improve the OS and/or PFS when used for the treatment of several tumors such as metastatic colon–rectal cancer, advanced non-small-cell lung cancer, or metastatic renal cell carcinoma, the introduction of anti-angiogenic therapies for the management of GBM patients, although initially promising, turned out to be only a “meteor” [46,47,48,49,50,51,52,53,54]. Apart from tumor recurrence or acquired resistance to angiogenic inhibitors, some evidence paradoxically reports a worsening in GBM aggressiveness after bevacizumab therapy [55]. The marginal clinical benefits may be attributed to the tumor resorting to alternative modes of neovascularization, aside from canonical angiogenesis, eventually exacerbated or activated as a compensation strategy by anti-angiogenic drugs. GBM harbors an exceptional level of intra- and inter-tumor heterogeneity as well, which poses further challenges in treatment. With the advances of groundbreaking research, three distinct molecular subtypes have been identified, namely classical (CL), proneural (PN), and mesenchymal (MES), characterized by different genetic backgrounds, prognoses, and therapeutic sensitivity [56]. Presumably, distinct subtypes exploit alternative neovascularization strategies, pro-angiogenic factors, and escape routes in a temporally and spatially variable manner. GBM with a mesenchymal signature, known for its worst outcomes, appears to strongly activate the trans-differentiation mechanism, for example, compared to the other subtypes, as suggested by the higher angiogenic potential of MES-GBM cancer stem cells (GSCs) relative to those of the PN subtype [57,58,59]. Moreover, unlike the other phenotypes, the mesenchymal one shows the greatest upregulation of pro-angiogenic factors like VEGF, VEGFR1/2, and PECAM1 (a signaling molecule expressed specifically by the endothelium). Additionally, the MES vasculature exhibits a higher Ki-67 index than the PN and CL subtypes, indicating enhanced levels of endothelial proliferation [60]. Therefore, understanding the inter-patient heterogeneity associated with different modes of neovascularization, as well as the multitude of underlying molecular dynamics and actors involved, is crucial for contributing to the development of novel personalized therapies.

In the present article, we review the multiple modes of neovascularization observed in the GBM ecosystem so far, summarizing the key factors and principle pathways involved. We also emphasize the structural features of tumor vasculature, as the result of the complex interplay between different neovascularization mechanisms. Moreover, we provide an updated version of the emerging therapeutic scenario in the fight against GBM angiogenesis, highlighting the urgent need for a different and innovative therapeutic approach, based on the specific molecular profile of the patient, with the possibility of combining therapies to hit multiple pathways, also those involved in angiogenesis.

## 2. GBM Vascularization: Morphological and Functional Aspects

**Figure 1:**
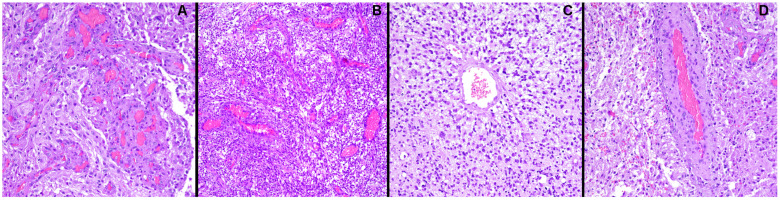
Some important morphological aspects of angiogenesis in GBM (E&E staining). (**A**) The image shows glomeruloid vessels (vessels with multiple lumina) and endothelial multilayering because of endothelial hyperplasia (200× magnification). (**B**) Atypical, branched vessel network (100× magnification). (**C**) An island of viable tumor cells encircling the blood vessels in a radial pattern (200× magnification). (**D**) Ectatic hyalinized vessels and intervening spindle-shaped stromal cells (200× magnification).

Angiogenesis, the generation of new blood vessels from pre-existing vascular networks, holds a pivotal role in both physiological and pathological settings, including cancer [61]. In order to grow, infiltrate the surrounding tissue, and eventually metastasize, tumors need an increased blood supply to meet the rising demand for nutrients, oxygen, and waste removal [62]. In this context, compared with other solid tumors, GBM has a marked angiogenic potential [63]. Interestingly, however, tumor-associated blood vessels are characterized by various morphological and functional abnormalities, deserving of a deeper evaluation (Figure 1) [64]. Neovascularization in GBM is often described as accelerated yet dysfunctional [65,66]. In depth, newly sprouted blood vessels appear dilated, fenestrated, and highly permeable; have a tortuous course; and give rise to a poorly branched and disorganized tree-like structure. Sometimes, immature or dead-end blood vessels are also formed [67,68]. In addition, GBM vasculature does not present the classical hierarchical organization into arterioles, venules, and capillaries [69]. Moreover, “glomeruloid microvascular proliferations”—vascular clusters made of hyperplastic endothelial cells, smooth muscle cells, and pericytes—are also present, commonly near necrotic foci [68].

At a single-cell level, GBM-associated endothelial cells (GECs) reveal larger sizes, larger nuclei, multiple nucleoli, and a flattened, veil-like appearance, unlike the cobblestone-shaped ones typically found in normal brain tissue [70,71]. GECs are also more resistant to apoptosis and migrate faster, exploiting chemo-kinesis instead of chemotaxis [70]. This blood vessel dysmorphia ultimately causes several functional defects, leading to increased regional hypoxia, a non-homogenous blood supply, enhanced permeability, and possible edema formation along with increased interstitial fluid pressure which overall hinders effective drug delivery [69,72,73,74]. Actually, a broad spectrum of different neovascularization patterns participates in the creation of such a particular vascular bed [75].

## 3. Different Strategies of Neovascularization in GBM

Sprouting angiogenesis represents the canonical process by which solid tumors build new blood vessels. However, advances in research have identified several alternative mechanisms implicated at various levels in tumor growth, progression, and drug resistance [76]. Currently, to what extent each mechanism plays a role in human brain cancer remains a controversial issue, as well as their involvement in the development of resistance to anti-angiogenetic drugs, as supported by clinical evidence [77,78]. Understanding the relative contribution in the context of GBM would lead to a deeper comprehension of the biology of angiogenesis. Recognizing how the various mechanisms interact (if they work together or sometimes antagonize each other or are occasionally activated as a compensatory mechanism) and what the underlying regulatory pathways are is a necessary step in designing more effective therapeutic approaches [76,79].

To date, (1) sprouting angiogenesis, (2) vasculogenesis, (3) vessel co-option, (4) vascular intussusception, (5) vessel mimicry, and (6) the trans-differentiation of cancer stem-like cells are all strategies that GBM can exploit to ensure a proper “fuel supply” [80]. Vessel co-option and vessel mimicry, in particular, have recently emerged as important players in several tumor types, as they are involved in both metastasis and resistance mechanisms to vascular-directed therapies [78,81,82].

### 3.1. Sprouting Angiogenesis

Angiogenesis, more specifically termed sprouting angiogenesis (SA), is a dynamic but strictly monitored process in time and space, which ensures the formation of vascular branches from pre-existing blood vessels when required, maintaining regular homeostasis [83,84]. Insufficient angiogenesis can lead to chronic wounds, ischemia, or hair loss, while excessive angiogenesis is often associated with eye disease, rheumatoid arthritis, or cancer [85].

SA represents the principal mechanism by which neovascularization occurs after birth, even if in the healthy adult it becomes largely quiescent [85,86]. The process turns on/off based on a delicate balance between pro- and anti-angiogenic factors [85]. Numerous mechanical (increasing blood flow, shear stress), chemical (hypoxic conditions, increased levels of nitric oxide), and molecular (growth factors, chemokines CXC-1, -2, -3, -5, -6, -7, and -8, hypoxia-inducible factor, integrins, and others) signals can trigger angiogenesis [87]. On the other hand, there exist main endogenous angiogenesis inhibitors (endostatin, angiostatin, chemokines CXC-4, -9, -10, -11, -12, and -14, thrombospondin, pigment epithelium-derived factor, interferon-α, -β, and -γ), often peptides or protein fragments present in the bloodstream or confined to the extracellular matrix [88,89].

**Figure 2:**
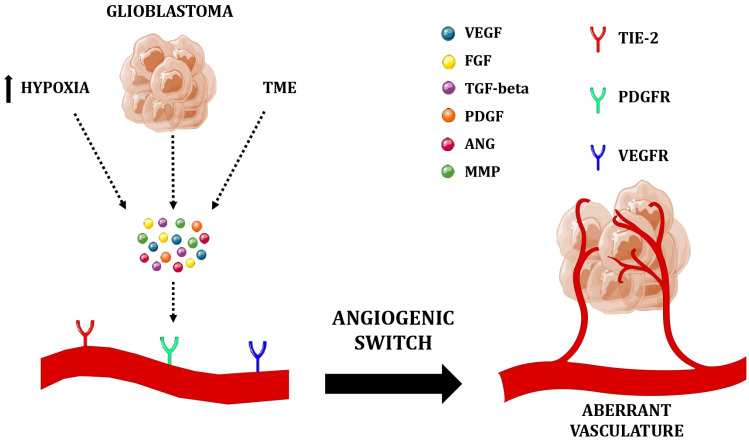
Schematic representation of the “angiogenic switch” in GBM. During tumor progression, the needle of the scale shifts in favor of pro-angiogenic factors, released by GBM cancer cells and TME, under hypoxic conditions, resulting in aberrant growth of GBM vasculature. Abbreviations: GBM, glioblastoma; TME, tumor microenvironment; VEGF, vascular endothelial growth factor; FGF, fibroblast growth factor; TGF-beta, transforming growth factor-beta; PDGF, platelet-derived growth factor; ANG, angiopoietin; MMP, matrix metalloproteinase; PDGFR, platelet-derived growth factor receptor; VEGFR, vascular endothelial growth factor receptor. This figure was created with Servier Medical Art (https://smart.servier.com).

Angiogenesis is a complex and multi-step process structured as follows: (1) angiogenic input; (2) degradation of extracellular matrix and dissolution of capillary basement membrane and activation of quiescent endothelial cells; (3) proliferation and migration of endothelial tip cells (cells located at the tips of the growing vessel that migrate following the VEGF concentration gradient); (4) tubulogenesis (the formation of an endothelial tube-like structure); and (5) the formation of a mature basement membrane with pericytes [83,90,91]. In GBM, with the “angiogenic switch” (Figure 2), the transition from dormant, avascular hyperplasia to the characteristic highly vascularized tumor with exuberant angiogenesis, the balance is disrupted and tips in favor of the pro-angiogenic factors [75,92]. Blood vessel sprouting, caused by an increase in angiogenesis activators, is promoted by hypoxic microenvironments, genetic alterations, and crosstalk between different cell types [63,83].

The key players and the principle molecular signaling pathways orchestrating abnormal angiogenesis in GBM are specifically described below.

#### 3.1.1. Hypoxia and Hypoxia-Inducible Factors

The hypoxic microenvironment—low tumor oxygenation—is the main driver of GBM angiogenesis. In the early stages, the tumor mass, being smaller than 1 mm, takes the necessary oxygen and nutrients from the pre-existing tissue vasculature [93]. However, beyond this size, in line with Folkman’s theory, the resulting hypoxic tumor ecosystem instigates the formation of new blood vessels through a series of genetic changes, necessary to feed the rapidly growing malignancy [93,94].

Hypoxia-inducible factor-1 (HIF-1) is the key mediator of cellular adaptation to hypoxic stress [95]. HIF-1 is a ubiquitously expressed transcription factor whose biological activity revolves around the formation of a heterodimer comprising the oxygen-labile α-subunit and the stable nuclear β-subunit [96]. Under normoxic cellular conditions, cells by default continuously produce but also rapidly degrade HIF1-α. In particular, with good oxygen levels, the prolyl hydroxylase domain (PHD) 1-3 hydroxylates HIF-α at the two proline residues 402 and 564 within the oxygen-dependent degradation domain (ODDD). The hydroxylated HIF1-α is then recognized and polyubiquitinated by Von Hippel–Lindau (VHL) E3 ubiquitin ligase and thus marked for proteasomal degradation. In contrast, under hypoxic conditions, HIF1-α is stabilized and accumulates in the cellular nucleus, where it forms a heterodimer with the β-subunit. Through binding to hypoxia response elements (HREs), HIF-1 ultimately modulates the expression of numerous target genes [95,96]. In GBM, due to the robust proliferation index and the aberrant angiogenesis, a highly hypoxic environment typically occurs [97]. In this context, HIF promotes the transcription of several pro-angiogenic factors, including VEGF, PDGF, angiopoietins, and their cognate receptors, as well as MMPs [95]. High levels of HIF-1 positively correlate with GBM development, metastasis, systemic dissemination, angiogenesis, and resistance to treatments [98,99]. Sometimes, in GBM, HIF can be activated even in normoxic conditions in the case of *EGFR* amplifications/mutations or in the case of *PTEN*/*TP53* loss [99].

#### 3.1.2. Pro-Angiogenic Factors

*VEGF*. Vascular endothelial growth factors (VEGFs) portray a family of homodimeric glycoproteins (molecular weight of about 40 kDa) with a cystine-knot motif that includes seven different members: VEGF-A, VEGF-B, VEGF-C, VEGF-D, the placental growth factor (PIGF), viral VEGF-E, and snake venom VEGF-F [100,101,102]. These factors trigger signal transduction cascades, binding to a three-member family of tyrosine kinase receptors with different specificity: vascular endothelial growth factor receptor-1 (VEGFR-1), VEGFR-2, and VEGFR-3 [103]. VEGF-R1 binds to VEGF-A and -B and PIGF, and VEGF-R2 is specific to VEGF-A, -C, and -D, while VEGF-R3 selectively binds to VEGF-C and -D. Furthermore, VEGF receptors perform their biological functions by interacting with different co-receptors, such as neuropilin-1 and -2 and heparan sulfate proteoglycans [104].

Researchers’ attention has mainly focused on the multifunctional cytokine VEGF-A, the most representative regulator of both normal and pathological angiogenesis [105,106,107].

Within a tumor context, various cell types secrete VEGF-A, including cancer cells but also endothelial cells, macrophages, and fibroblasts [107]. Although VEGF-A binds to both VEGFR-1 and VEGFR-2, VEGF-A/VEGFR-2 is the dominant signaling pathway [108]. Several factors produced in response to hypoxic tumor microenvironments (HIFs, PDGF, EGF) or independently of hypoxia can induce VEGF and VEGFR upregulation in GBM [54,108,109]. In particular, high levels of VEGF-A mRNA were detected, especially near necrotic areas in GBM tumors [110]. Furthermore, the VEGF-A expression rate increases from low-grade to high-grade gliomas and negatively correlates with GBM patients’ survival [111,112]. Moreover, GBM exhibits the highest content of VEGF protein, compared with other brain tumors, such as ependymomas, meningiomas, and medulloblastomas [113]. Triggering signaling pathways (such as PI3K/AKT/mTOR, RAS/RAF/MEK/ERK, SRC/FAK, and p38 MAPK), VEGF-A/VEGFR-2 promotes vascular permeability as well as EC proliferation and migration. However, besides stimulating tumor angiogenesis, VEGF-A is also implicated in the growth, survival, and infiltration of tumor cells, particularly in aggressive tumors. Although VEGF mainly acts through paracrine mechanisms by binding to receptors expressed on the tumor vasculature, GBM cancer cells are also able to express VEGFRs, stimulating malignancy progression through autocrine signaling [54,92,113,114]. Furthermore, it has been observed that VEGF-A promotes tumorigenesis and angiogenesis in human GBM cancer stem-like cells, though it is still unclear whether it also stimulates their proliferation and through what mechanisms [115]. The alternative splicing of VEGF-A pre-mRNA allows for the production of various protein isoforms, characterized by different molecular weights, biochemical properties, and biological functions. VEGF-121, the lighter variant with no heparin-binding domain, is the main isoform in circulating blood, believed to be predominantly involved in vascular permeability and less involved in tumor angiogenesis [116,117]. The heavier isoforms, VEGF-A189 and VEGF-A206, are closely anchored to the extracellular matrix by their two heparin-binding domains, serving as a valuable source of VEGF [116,118,119]. Instead, the intermediate-weight VEGF-A165 isoform, which has a single heparin-binding domain, presents in-between ECM-binding properties, showing excellent bioavailability [116,118]. As recently suggested by D’Alessandris et al., GBMs can synthetize all VEGF isoforms. Therefore, since bevacizumab binds to all VEGF splicing products, the sensitivity of each GBM tumor to anti-angiogenic treatment may depend on the relative amount of the various isoforms produced [120]. Martini and colleagues, using a brain xenograft model of human GBM cells, observed that VEGF-A121 plasma levels correlate with tumor size and that VEGF-A121 concentration in peripheral blood markedly decreases after bevacizumab administration. Additionally, if higher levels of circulating VEGF-121 may result in less bevacizumab being available to target the other, more clinically relevant VEGF isoforms, the research group demonstrated that patients with lower baseline VEGF-121 concentrations and lower VEGF-121 reduction after bevacizumab treatment had better prognoses. Therefore, the circulating VEGF-A121 levels could be a potential predictive biomarker for response to bevacizumab in GBM patients [116].

Apart from VEGF-R2, VEGF-R1 under physiological conditions can prevent excessive blood vessels sprouting by acting as a negative regulator, but when upregulated in tumors, it seems to contribute to aberrant angiogenesis [121]. VEGFR-3, instead, normally mediates lymphoid proliferation, but evidence suggests its involvement in tumor metastases. However, more investigations are required in this thematic area [121].

*FGF*. The human genome encodes 22 polypeptides (molecular weights range from 17 to 34 kDa) that form the broad family of fibroblast growth factors (FGFs), with FGF1 (acid FGF) and FGF2 (basic FGF) being the first members to be identified in the 1970s [122,123,124,125]. FGFs mediate the behaviors (proliferation, differentiation, migration, survival) of various cell types, tissue maintenance, and wound healing through fibroblast growth factor receptor (FGFR) activation [124,126,127]. However, improper FGF/FGFR signaling is also implicated in cancer progression and tumor-induced angiogenesis [128]. Previous studies have widely highlighted FGF upregulation in various tumor contexts [129,130,131,132,133,134,135]. In glioblastoma cases, specifically, increased levels of both FGF2 mRNA and proteins have been reported through Northern blotting, immunohistochemistry, and in situ hybridization.

Moreover, the expression rate of FGF2 was positively correlated with tumor aggressiveness and vascular density [136,137]. Compelling evidence, in fact, shows that FGF2 plays a crucial pro-angiogenic role in GBM [138]. Basically, FGF2, in synergy with VEGF, stimulates endothelial cell replication and migration as well as the proteolysis of the extracellular matrix, necessary for the formation of new blood vessels [139,140,141,142]. Interestingly, FGF inhibitors have shown anti-angiogenic effects in murine models, also leading to an increase in tumor cell apoptosis [143]. Indeed, it has been observed that FGF2 can promote glioma cell survival by upregulating BCL2 and seems to support, together with EGF, the self-renewal of GBM stem-like cells [144,145]. In addition, an in vitro study by Toyoda et al. demonstrated how FGF2, secreted by GBM cells, can improve blood–tumor barrier functionality, contributing to the typical GBM refractoriness to therapies [146]. Furthermore, aberrant FGF2/FGFR signals are also involved in acquired resistance to anti-VEGF drugs in GBM patients, but the related mechanisms remain to be clarified [147].

FGFRs comprise a group of five transmembrane tyrosine kinase receptors, from FGFR1 to FGFR5 (FGFR5 is a truncated receptor, lacking the cytoplasmic tyrosine kinase domain) [148]. Cognate ligand/receptor interactions trigger various intracellular molecular pathways, including RAS/MAPK, PLCγ, PI3K-AKT, and STATs [149,150,151,152]. However, approximately 8% of gliomas harbor FGFR genomic aberrations in the form of activating mutations, amplifications, and structural rearrangements, which contribute to tumor progression. *FGFR-TACC* fusion and *FGFR1*/*FGFR3* amplifications constitute the most common variants [153,154,155].

*TGF-β*. The three well-characterized transforming growth factor-*β* (TGF-β) isoforms (TGF-β 1, -β 2, and -β 3) are 25 kDa cytokines with pleiotropic effects, secreted as inactivate precursors almost ubiquitously in the human body [156]. TGF-βs, by activating three specific membrane receptors (TβRI, TβRII, TβRIII), trigger both Smad-dependent and Smad-independent molecular pathways (PI3K/AKT, RHO, PAR6, ERK, JUNK, p38, NF-κB, and TRAF4/6), leading to different, context-dependent, cellular responses [156,157,158]. Intriguingly, TGF-β shows a dualistic behavior in tumorigenesis, acting as a cancer repressor in healthy tissues/premalignant stages but also turning into a strong promoter of tumor progression and malignancy in advanced disease [159]. Specifically, dysregulated TGF-β signals drive more aggressive tumor phenotypes supporting angiogenesis, infiltration, metastasis, immunosurveillance evasion, and epithelial-to-mesenchymal transition programs [159,160,161,162,163,164,165]. Even in GBM, the so-called “TGF-β paradox” has been widely observed [166,167]. Increased TGF-β has been found in the most aggressive gliomas and represents a poor prognostic marker, both in terms of the PFS and OS [167]. TGF-β, secreted by cancer cells or other cell types in the surrounding microenvironment, also encourages GBM neovascularization, mainly by augmenting the levels of the pro-angiogenic factor insulin-like growth factor-binding protein 7 (IGFBP7) in the tumor endothelial fraction [168]. Furthermore, impaired TGF-β signaling indirectly stimulates endothelial cell proliferation and ECM remodeling in GBMs, increasing VEGF production and MMP functionality, respectively [169,170]. Additionally, cell proliferation through TGF-β/Smad has been highlighted in human glioma cell lines. Indeed, apart from TGF-β, phospho-Smad overexpression has been demonstrated by in vivo studies [171].

*PDGF*. Initially isolated from human platelet α-granules but also released by other cell types, platelet-derived growth factor (PDGF) acts as a potent stimulator of fibroblast, glial cell, and smooth muscle cell proliferation, apart from being involved in migration and chemotaxis [172,173,174,175,176]. The PDGF family includes four protein chains (PDGF-A, PDGF-B, PDGF-C, PDGF-D) that can variously combine to generate disulfide-bonded homodimers (PDGF-AA, PDGF-BB, PDGF-CC, PDGF-DD) and heterodimers (PDGF-AB), mediating all the biological effects ascribable to PDGF, through PDGFR activation [177,178]. Platelet-derived growth factor receptors (PDGFR-α and PDGFR-β) are single-pass membrane tyrosine kinase glycoproteins with a molecular weight of approximately 180 kDa [179].

After proper stimulation by the ligand, PDGFRs trigger multiple downstream signaling cascades, including PI3K/AKT, JAK/STAT, the Notch pathway, and MAPK/ERK [180,181]. Aberrant PDGF signaling networks show tumorigenic potential in a variety of tumor forms, contributing to progression, metastasis, angiogenesis, the EMT, and anti-VEGF escape [182,183,184,185]. Both ligands and their receptors were found to be upregulated in gliomas. About 20% of GBMs exhibit genetic alterations in *PDGF*/*PDGFR* signaling, commonly *PDGFs* activating mutations/chromosomal rearrangements or *PDGFR* amplifications/overexpression [182,186,187]. Mounting evidence demonstrates that PDGF-B is the main factor involved in promoting GBM angiogenesis by stimulation through its receptor PDGFR-β, pericyte recruitment, and increasing VEGF levels in the developing tumor vasculature [188]. Furthermore, in GBM tissues, in situ hybridization analyses have revealed the enhanced expression of both PDGF-B and PDGFR-β mRNA in the proliferating endothelial component, particularly in glomeruloid structures and small capillaries, suggesting the existence of autocrine angiogenesis-stimulating loops [189]. GCSCs express PDGFR-β more frequently than PDGFR-α. In GBM cell lines, the genetic or pharmacological inhibition of PDGFR-β has been proven to cause a lowering of GSCS self-renewal potential and a decrease in GBM progression [186]. Instead, PDGFR-α targeting in vitro promotes apoptosis in GCSCs resistant to anti-Notch/anti-EGFR therapy [190]. It has also been suggested that PDGF-α/β inhibition with CP-673451 both attenuates proliferation/invasion rates and stimulates differentiation in GBM/GCSCs cell lines via DUSP1/p38 MAPK [191].

*Angiopoietins.* Originally discovered in 1996, angiopoietins constitute a small class of four endothelial growth factors, angiopoietin-1 (Ang1), Ang2, Ang3 (in mouse), and Ang4 (in humans), which structurally share an amino-terminal “superclustering” region, followed by a coiled-coil motif involved in multimerization and a carboxy-terminal fibrinogen-like domain necessary for receptor binding [192,193,194]. All angiopoietins act through Tie-2 (also known as TEK), a tyrosine kinase receptor specifically expressed on endothelial cells and their precursors [195,196,197]. The Tie family also includes Tie-1, a poorly defined orphan receptor that presumably affects Ang1-Ang2/Tie-2 signaling, modulating both physiological and pathological neovascularization [198,199].

Ang1 is a natural TEK agonist, essentially involved in vascular maturation and quiescence, whereas Ang2 functions as a context-specific agonist or antagonist of TEK, promoting angiogenesis or blood vessel destabilization/cell death in the presence or absence of VEGF-A, respectively [193,200,201]. Interestingly, the angiopoietin/Tie2 ligand/receptor system is frequently perturbed in cancer patients. It has been remarked that an altered expression profile of Angs can intensify the aggressiveness of ovarian cancer, lung carcinoma, and human gliomas, while their contribution to tumor angiogenesis is not well defined [202,203,204,205]. Various studies conducted on this research topic have revealed conflicting data, reporting, in some cases, pro-angiogenic roles and, in other cases, anti-vascular effects of upregulated Ang1 or Ang2 [206]. In GBM, the Ang/Tie-2 axis presumably contributes to aberrant angiogenesis [206]. Furthermore, Northern blot and in situ hybridization analyses showed the differential expression of Ang ligands in human GBM tissue samples: the upregulation of Ang1 was found in tumor cells, while Ang2 mRNA was detected only in the endothelial cells within the tumor mass but exclusively in correspondence with small capillaries. In addition, immunohistochemistry indicated enhanced levels of Tie-2 receptor in tumor vasculature, compared to in healthy brain tissue, in a rat glioma model [207]. Moreover, apart from endothelial cells, GBM tumor cells are also capable of expressing the Tie-2 receptor [208]. The exact biological significance of this imbalanced pathway in GBM has yet to be elucidated. However, it has been observed that a double pharmacological blockade of VEGF and Ang2 in two murine GBM models (Gl261 and U87) allowed the normalization of tumor vasculature and interfered with malignancy progression, leading to better prognoses [209]. Other experts have highlighted that the protein levels of Ang4 are also enhanced in human GBM tissues. Being implicated in tumor angiogenesis and development, it could be a potential future therapeutic target [208].

#### 3.1.3. Proteinases

*MMPs.* The family of matrix metalloproteinases (MMPs) includes a large collection of endopeptidases with different substrate specificity, which specifically require zinc and calcium to perform their enzymatic activity [210]. Described for the first time in the tadpole, MMPs have rapidly emerged as the group of proteases mainly involved in ECM remodeling [211]. Collectively, by cleaving internal peptide bonds, MMPs are able to break down the majority of protein components constituting connective tissues such as collagen, elastin, fibronectin, gelatin, and casein [211]. ECM degradation is a crucial step both during embryonic development and in postnatal life. However, apart from mediating several physiological processes, dysregulated MMP activities are commonly found in pathological states, including tumor neovascularization, invasion, and spread [212,213].

Basically, MMPs augment angiogenesis through multiple mechanisms spanning from increasing the bioavailability of ECM-bound pro-angiogenic mediators to facilitating endothelial cell migration and pericyte detachment from growing vessels [214].

Comparing different cancer types, RNA-seq analyses have recently outlined a highly heterogeneous MMP expression, showing overall enhanced levels of MMP1, MMP9, MMP10, MMP11, and MMP13 but the downregulation of MMP2, MMP7, MMP23B, MMP27, and MMP28 [213]. The potential role of MMPs in glioma pathogenesis has also been investigated. In this respect, accumulating data have highlighted an increased expression of MMP1, MMP-9, MMP-11, and MMP-19 in GBM tissues, both at the mRNA and protein levels. Upregulated MMP-1 and MMP-9 negatively correlate with the patient’s prognosis, while MMP-11 overexpression seems to be associated with a more aggressive GBM phenotype [215,216]. Furthermore, it has been observed that an unbalanced MMP9 activity elevates the rate of cell proliferation and the clonogenic potential in U87 GBM cell lines [217]. Moreover, a randomized phase III study has shown that newly diagnosed GBM patients with low MMP9 plasma levels respond better to bevacizumab, with an improved OS, reinforcing the hypothesis of its concrete involvement in GBM angiogenesis [218]. The therapeutic value of conventional chemotherapy combined with a broad-spectrum MMP inhibitor in the treatment of GBM has recently been studied in some clinical trials, with encouraging data [219].

### 3.2. Vasculogenesis

During embryo development, the primitive vascular system originates from the ex novo assembly of endothelial precursor cells derived from the mesoderm, during a process known as vasculogenesis [220,221]. For a long time, it has been considered a mechanism exclusive to the intrauterine period [222]. Even in adult life, in reality, different scenarios require vasculogenesis activation like wound healing, stroke, ischemia, or tumor development [223]. Bone marrow, hematopoietic stem cells, myeloid cells, and tissue-resident cells (adipose, cardiac, dental, or neural tissue) can all be putative sources of adult endothelial progenitor cells (EPCs). Subsequent to GBM’s recruitment of EPCs, they predominantly occupy the hypoxic niches and start to differentiate into mature endothelial cells, initiating the formation of new blood vessels [222,224,225]. In GBM patients, as with other tumor types, the circulating levels of EPCs were found to be elevated compared to in healthy individuals. Furthermore, it has been shown that an increase in glioma malignancy grade is positively correlated with a higher fraction of VEGF-R2+ cells [226]. Moreover, EPC recruitment, which appears more prominent in GSC-enriched GBM tumors, is also further intensified after radiotherapy treatment. In a radiotherapy-treated GBM xenograft model, it was observed that the tumor was able to recover its damaged endothelial fraction through the induction of vasculogenesis. Both HIF-1 (activated by hypoxia and reoxygenation after radiotherapy) and stromal cell-derived factor-1 (SDF-1), along with its receptor CXCR4, play a crucial role in recruiting bone marrow-derived cells (BMDCs) to the tumor [227,228,229,230]. Through the pharmacological targeting of HIF-1 or the inhibition of CXCR4 with neutralizing antibodies, the influx of BMDCs was interrupted, contrasting tumor recovery. These results reiterate the need to target GBM on multiple fronts, also considering the existence of vasculogenesis as a compensatory mechanism of neovascularization exploited by the tumor [231].

### 3.3. Vessel Co-Option

That cancerous cells can co-opt a pre-existing vascular system has been demonstrated, for the first time, in a rat C6 glioma model. Tumors affecting the brain, liver, and lungs, organs characterized by a high vascularization level, show the phenomenon more commonly. Specifically, during blood vessel co-option, tumor cells, given their infiltrating ability, penetrate the healthy tissue and migrate along the pre-existing capillaries, eventually wrapping them and guiding them into the tumor mass so that host vasculature-dependent proliferation can start [82,232]. Recent studies suggest that the vessel co-option (VC) mechanism can be intrinsic to the tumor or eventually exacerbated by anti-angiogenetic therapies/chemoradiotherapy, allowing GBM to recur inexorably [233,234].

There is currently no detailed information on the vessel co-option modus operandi due to the challenges related to the longitudinal observation of VC in vivo and the lack of adequate in vitro models [235]. Ang-2, IL-8, CDC42, CXCR4/SDF1α, bradykinin, ephrinB2 (only when upregulated), and Olig2/Wnt7a have emerged as some of the key players in driving GBM vessel co-option [234].

### 3.4. Vascular Intussusception

Vascular intussusception (VI) takes place when a pre-existing blood vessel splits into two functional blood vessels. Vascular remodeling occurs through blood vessel invagination followed by the formation of intraluminal intussusceptive pillars that ultimately create a double lumen [77,79]. The existence of intussusceptive microvascular growth as one of the potential backup mechanisms to ensure tumor perfusion has emerged in GBM but also in melanoma, colon cancer, breast carcinoma, and B-cell non-Hodgkin’s lymphoma [236]. An angiogenic switch from sprouting to intussusception associated with malignancy recovery has been reported after ionizing radiation or anti-VEGF treatment (PTK787/ZK222854) in tumor xenografts in nude mice [237]. Compared to canonical neovascularization, VI requires less energy and time consumption [236]. In addition, newly formed blood vessels are less permeable and appear more refractory to anti-angiogenic inhibitors. Every aspect is particularly useful for GBM progression [236,238]. The mediators leading this process have not yet been identified [77].

### 3.5. Vasculogenic Mimicry

Under certain conditions, GBM aggressive tumor cells, marked by high plasticity, organize spatially to form vessel-like channels that are then connected with the pre-existing vascular system in order to provide the tumor mass with enough nutrients and oxygen [239,240]. First discovered in melanoma, two different vascular mimicry (VM) modalities have been identified in tumors so far: the vasculogenic mimicry of the patterned matrix mode and the vasculogenic mimicry of the tubular mode [239,241]. In the first type, the channels have a completely distinct morphology compared to endothelial blood vessels, while in the second case, vasculogenic-like structures closely resemble capillaries [241]. Both strategies have been observed in GBM, and the incidence of VM is often associated with resistance to anti-angiogenic drugs and worse prognoses [240,241,242,243,244]. Indeed, the research group of Angara et al. has previously outlined increased VM in GBM following anti-angiogenic treatments (Vatalanib), supporting the hypothesis that it is an alternative neovascularization mechanism, mainly induced by hypoxic conditions but aggravated by angiogenesis inhibitors [239,245]. The so-called tumor-derived endothelial cells (TDECs)—mature GBM tumor cells trans-differentiated into cells with an endothelial phenotype—play a key role in promoting VM [246].

### 3.6. Trans-Differentiation of Cancer Stem-Like Cells

Recent research efforts have highlighted how the aggressive behavior of GBM is largely attributable to GBM stem-like cells (GSCs), a small fraction of cancerous cells, with high plasticity and self-renewal capacity, involved in tumor initiation, maintenance, and progression [247,248]. Cancer stem-like cells (CSCs) were first identified at the end of the 20th century. Bonnet and Dick, indeed, were pioneers in isolating CSCs from acute myelogenous leukemia [249]. Since then, CSCs have become a hotspot on which many research groups have focused. Their discovery has certainly provided new insights into understanding tumor biology and eventually finding novel molecular biomarkers.

GSCs, typically located near hypoxic perivascular niches, show a slow-growing status and a marked resistance to conventional treatments (both chemo- and radiotherapy) [250]. Endowed with stemness properties, GSCs exhibit a high differentiation potential, causing extensive tumor heterogeneity, a hallmark of GBM [250]. It is now widely accepted that GSCs can also trans-differentiate into an endothelial lineage, directly contributing to new blood vessel formation [251]. In support of this, Ricci-Vitiani et al. found that, on average, 60% of GBM endothelial cells showed the same chromosomal aberrations as those reported in GBM tumor cells, suggesting a cancerous origin [252]. Furthermore, they observed trans-differentiation both in vitro, with the isolation of CD133+/CD31− GBM cells capable of obtaining an endothelial phenotype, and in vivo using a xenograft model [253].

Moreover, the work of De Pascalis et al. has shown that in GBM recurring after radiotherapy, the cerebral endothelium undergoes radiation-induced senescence, and the fraction of tumor-derived cells with an endothelial phenotype increases by a factor of 2 to 2.7 compared to tumors from primary surgery. Therefore, GSCs likely play a compensatory role by supporting the neovascularization of the brain endothelium undergoing radiation-induced senescence [254]. This GSC-associated neovascularization mechanism seems closely connected to hypoxia and occurs independently of the VEGF/VEGFR signaling pathway. Therefore, bevacizumab, or other drugs that target the same pathway, have not proven to be very effective at blocking the trans-differentiation of GSCs into GSC-derived endothelial cells (GdECs) [253]. However, the efficacy of 349 different compounds in inducing cell death in both GSC and GdEC cell lines was comprehensively evaluated, with elesclomol (STA-4783) emerging as the most effective. Elesclomol compromises the survival of both GSCs and GdECs, inducing a significant increase in mitochondrial reactive oxygen species, leading to non-apoptotic, copper-dependent cell death. Furthermore, as confirmed both in vitro and in vivo, the combination of elesclomol and TMZ enhances cytotoxicity compared to TMZ alone [255].

## 4. Treating GBM: Between Pitfalls and Emerging Therapies

The current lack of successful therapies represents one of the major limitations in GBM management. Several aspects contribute to making treatment options insufficient. Firstly, removing the entire tumor is tricky given its critical location and highly infiltrative nature. Even when the mass appears completely excised, GBM invariably comes back. Furthermore, the presence of the blood–brain barrier (BBB) significantly obstructs effective CNS drug delivery [256]. Most GBM patients are also intrinsically refractory to chemo-/radiotherapy or develop resistance at a later time, a phenomenon primarily driven by CSCs [257,258]. In addition, both the great intra-tumor heterogeneity and the marked immunosuppressive microenvironment are closely related to the failure of current treatments [259,260]. Moreover, aberrant angiogenesis and the coexistence of multiple modes of neovascularization complicate the effectiveness of anti-angiogenic therapies [261].

The therapeutic landscape in the GBM setting is, however, currently evolving, and immense efforts have been directed towards overcoming the Stupp protocol, the gold-standard treatment to date, through the development of more promising therapeutic approaches [262].

Bevacizumab (BEV), or Avastin, is a recombinant monoclonal IgG1 antibody, humanized, specifically directed against VEGF-A, typically upregulated in GBM [63,263,264,265]. Based on data from two phase II studies, BRAIN and NCI 06-C-0064E, it was FDA-approved in 2009 as a single agent for the treatment of recurrent glioblastoma, but its concrete ability to improve the prognosis remains to be clarified [266,267]. In particular, in the BRAIN trial, 167 patients with GBM in their first or second relapse were randomly assigned to receive BEV, alone or in combination with Irinotecan (a topoisomerase I inhibitor). The 6-month PFS rates were 42.6% and 50.3%, respectively, and the median OS rates were 9.2 months and 8.7 months, respectively. Moreover, the group receiving BEV plus Irinotecan experienced an increased incidence of grade 3 or higher treatment-related adverse events compared to the BEV-alone group [268]. In the 06-C-0064E study, a single-center experience conducted by the National Cancer Institute (NCI), instead, a cohort of 48 patients with recurrent GBM was treated with BEV monotherapy until progression, resulting in a 6-month PFS of 29% and a median OS of 31 weeks [269].

Given the single-agent treatment failure, the spotlight has shifted to the possible application of polytherapy. The combination of two or more anti-cancer drugs, indeed, has recently emerged as a novel promising angle to explore, aiming to better prognoses and increase therapeutic potential while reducing side effects [270].

In this respect, different clinical trials have examined the efficacy and toxicity of BEV together with other chemotherapeutic drugs or radiotherapy, highlighting improvements in the OS and/or PFS [271,272,273,274]. For example, the potential of BEV or Lomustine as single agents versus a combination of BEV plus Lomustine was evaluated in a group of 153 patients with recurrent GBM, in a randomized controlled phase II study called the BELOB trial. The primary end point was a 9-month OS. The main secondary end points were the median PFS, median OS, objective response rate, and association of the outcome with MGMT (O^6^-methylguanine-DNA methyltransferase) promoter methylation status and IDH status. Overall, the 6-month PFS and the median OS were 16% and 8 months in the BEV group, 13% and 8 months in the Lomustine group, and 42% and 12 months in the BEV plus Lomustine group, respectively. The safety analysis showed that, after a reduction in the Lomustine dose, the combination treatment was generally well tolerated, suggesting promising prospects for the use of BEV integrated with Lomustine [273]. Later, following the findings of the BELOB study, the EORTC (European Organization for Research and Treatment of Cancer) 26101 phase III trial enrolled a total of 437 patients with recurrent GBM, of whom 149 underwent Lomustine treatment alone, and 238 received Lomustine plus BEV for a period of 37 months. Despite a longer PFS in the group treated with the combination therapy (4.2 months vs. 1.5 months), there was no significant difference in the median OS between the two cohorts (9.1 months in the combination group and 7.6 months in the monotherapy group) [266,275]. More precisely, in the RTOG 0825 trial, 637 newly diagnosed GBM patients were randomly assigned to receive either BEV or a placebo, in addition to TMZ and radiotherapy. While the study suggested an improvement in the PFS (10.7 months in the BEV group compared to 7.3 months in the placebo group), it showed no evidence of a survival benefit. The median OS was, indeed, 15.7 months in the BEV group and 16.1 months in the placebo group, respectively [263]. Based on the same rationale, a second placebo-controlled study, called the AVAglio trial, enrolled a total of 921 newly diagnosed GMB patients who were randomly assigned to the BEV group (458 patients) or to the placebo group (463 patients). The addition of BEV to TMZ + radiotherapy prolonged the PFS (8.4 months in the BEV group, 4.3 months in the placebo group) and extended the baseline performance status, along with decreasing the glucocorticoid demand. Conversely, the duration of OS observed did not significantly differ between the two cohorts (16.8 months in the BEV group compared to 16.7 months in the placebo group) [276]. However, AVAglio patients were then retrospectively evaluated for molecular subtype, and the group with proneural GBM actually showed an increase in OS after treatment with Bevacizumab added to first-line therapy [277].

The potential role of alternative angiogenic inhibitors, including monoclonal antibodies (Carotuximab anti-endoglin), tyrosine kinase inhibitors involved in angiogenesis (Regorafenib: anti-VEGFR1,2,1, FGFR1, PDGFR-β, Sorafenib anti-VEGFR2/3, PDGFR-β, Raf kinase, and FLT3; Sunitinib: anti-VEGFR2, PDGFR-α/β, and KIT; Cediranib: a pan-VEGFR inhibitor; and others), and recombinant fusion proteins (Aflibercept, a decoy receptor anti-VEGF), either as monotherapy or in combination with other anti-cancer drugs, is being investigated in numerous ongoing phase I, II, or III clinical studies, widely described in the current literature with promising preliminary results [42,278,279,280,281,282,283,284,285,286,287,288].

The introduction of immunotherapy in clinical practice has changed the management of previously difficult-to-treat solid tumors. However, immunotherapy in GBM has not led to significant improvements in clinical outcomes. GBM is a cold tumor, capable of implementing various immune escape strategies [260,289]. Nivolumab (anti-PD-1), Pembrolizumab (anti-PDL-1), and Ipilimumab (anti-CTLA4) are among the main immune checkpoint inhibitors tested as single agents in several clinical trials against recurrent or newly diagnosed GBM but with marginal clinical benefits [260]. However, the therapeutic efficacy of immune checkpoint inhibitors combined with anti-angiogenic drugs or with vaccine-based/oncolytic virus-based/CAR-T-based approaches is currently under critical evaluation [260,290].

The details of the most relevant clinical trials involving anti-angiogenic drugs, used either alone or as part of a polytherapy regimen, in patients with recurrent or newly diagnosed GBM, are provided in Table 1.

Questions regarding the optimal dosing regimen of BEV and other emerging drugs for GBM management remain unresolved. While maximizing the likelihood of a response, it is equally important to minimize therapy-related toxicity. However, the persistently high incidence of drug-related side effects continues to be a significant limitation and requires further investigation. From this perspective, for example, a phase II, comparative, randomized, single-center clinical trial was specifically performed to compare the efficacy and safety of low-dose BEV (5 mg/kg) plus Lomustine versus standard-dose BEV (10 mg/kg) alone in 69 adults with recurrent GBM. The results showed a trend towards a longer median PFS in the low-dose BEV + Lomustine arm in patients with their first relapse [291]. 

Furthermore, the presence of the blood–brain barrier, due to its highly protective nature, complicates proper drug delivery to the brain, possibly resulting in suboptimal concentrations of therapeutic agents at the tumor site and necessitating the development of strategies to enhance drug penetration and reduce off-target effects [292].

Ultimately, one of the key challenges for the future will be the identification of effective tumor-specific biomarkers and their integration with the assessment of each GBM’s genetic signature, to predict which patient subgroups could benefit most from novel anti-angiogenic therapies, either alone or in combination with other drugs. In this context, new research avenues are currently exploring the potential of various angiogenic regulators as biomarkers (such as lower concentrations of VEGF-A121 as a predictive circulating biomarker for response to BEV or downregulated HIF1-α as a predictive tissue biomarker for response to TMZ), in addition to identifying potential GSC biomarkers (such as CD133, CD44, SOX2, CD15, CD70, ALDH3A1, and nestin) for the development of targeted and personalized therapies [116,293,294].

## 5. Conclusions

GBM is hallmarked by a singular vascular bed, in terms of morphology, architecture, and functionality, which reflects a complex underlying biology. Tumor aspects like multiple pro-angiogenic mediators and pathways, alternative strategies of neovascularization, high intra- and inter-tumor heterogeneity, and strong resilience to current treatments emphasize the imperative need to design novel standards of care in the fight against GBM, specifically tailored to each patient’s molecular signature, which may impact various aspects of multifaceted neovascularization. Could combination therapies, corroborated by a more detailed comprehension of GBM’s intricate landscape, represent valid options to improve patients’ clinical outcomes and their healthy lifespan in the near future?

## Figures and Tables

**Table 1:** List of the major clinical trials targeting angiogenesis in GBM, including information such as trial name, phase, number of patients, dose, progression-free survival (PFS), overall survival (OS), and treatment-related side effects.

Trial Name/ Author	Treatment	Phase	No. of Patients	Dose	6-Months PFS	Median OS	Adverse Effects
BRAIN [268]	BEV alone/ BEV+ Irinotecan	II	167 patients with recurrent GBM	10 mg/kg every 2 weeks of BEV alone or 10 mg/kg of BEV + 340 mg/m^2^ or 125 mg/m^2^ of Irinotecan (if taking or not taking enzyme-inducing antiepileptic drugs, respectively) every 2 weeks	42.6% BEV alone 50.3% BEV+ Irinotecan	9.2 months BEV alone 8.7 months BEV+ Irinotecan	Hypertension, fatigue, headache, convulsion, diarrhea, nausea, and neutropenia
NCI 06-C-0064E [269]	BEV	II	48 patients with recurrent GBM	10 mg/kg every 2 weeks	29%	31 weeks	Thromboembolic events, hypertension, hypophosphatemia, and thrombocytopenia
BELOB [273]	BEV/ Lomustine alone or BEV + Lomustine	II	153 patients with recurrent GBM	110 mg/m^2^ every 6 weeks of Lomustine alone or 10 mg/kg every 2 weeks of BEV alone or 90/110 mg/m^2^ every 6 weeks of Lomustine combined with 10 mg/kg every 2 weeks of BEV	13% Lomustine alone 16% BEV alone 42% BEV + Lomustine	8 months Lomustine alone 8 months BEV alone 12 months BEV+ Lomustine	Hypertension, fatigue, and infections
EORTC 26101 [275]	BEV + Lomustine or Lomustine alone	III	437 patients with progressive GBM	10 mg/kg every 2 weeks of BEV + 90 mg/m^2^ every 6 weeks of Lomustine or 110 mg/m^2^ every 6 weeks of Lomustine alone	Not available	9.1 months BEV + Lomustine 8.6 months Lomustine alone	Pulmonary embolism, arterial hypertension, and hematologic toxic effects
RTOG 0825 [263]	BEV/placebo + TMZ + radiotherapy	III	637 patients with newly diagnosed GBM	10 mg/kg every 2 weeks of BEV + TMZ + radiotherapy	Not available	15.7 months	Lymphopenia, neutropenia, fatigue, and thrombocytopenia
AVAglio [276]	BEV/placebo + TMZ + radiotherapy	III	921 patients with newly diagnosed GBM	10 mg/kg every 2 weeks of BEV + TMZ + radiotherapy	Not available	16.8 months BEV 16.7 months placebo	Hypertension, proteinuria, thromboembolia, and wound healing complications
ENDOT [278]	Carotuximab (TRC105) alone or TRC105 + BEV following radiation, TMZ, and BEV therapy	II	22 patients with GBM that had progressed after chemoradiation	10 mg/kg weekly TRC105 alone/ 10 mg/kg split into two doses with 3 mg/kg administered on cycle 1 day 8 and 7 mg/kg administered on cycle 1 day 11 with TRC105 + BEV	13.3% (calculated for the 15 evaluable patients treated with BEV + TRC105)	5.7 months (calculated for the 15 evaluable patients treated with BEV + TRC105)	Headache, epistaxis, fatigue, TIA, lower leg edema, pulmonary embolism, and sinusitis
REGOMA [286]	Regorafenib or Lomustine	II	119 patients with recurrent GBM	160 mg once daily for the first 3 weeks of each 4-week cycle of Regorafenib or 110 mg/m^2^ every 6 weeks of Lomustine	Not available	7.4 months in the Regorafenib group/ 5.6 months in the Lomustine group	Hand–foot skin reaction, increased lipase, increased blood bilirubin, decreased platelet/ lymphocyte count, and neutropenia
Hottinger, A.F [281]	Sorafenib + TMZ + radiotherapy	I	17 patients with newly diagnosed GBM	400 mg 2 times daily	86.7%	17.8 months	Thrombocytopenia, neutropenia, alopecia, nausea, vomiting, hypophosphatemia, and fatigue
STELLAR [287]	Sunitinib or Lomustine (in the control arm)	I	32 patients with recurrent GBM in part I 37 patients with recurrent GBM in part II	Part I 300 mg Q1W of Sunitinib or 110 mg/m^2^ of Lomustine once every six weeks Part II 700 mg Q2W of Sunitinib or 110 mg/m^2^ of Lomustine once every six weeks	8% in part I with Sunitinib vs. 29% with Lomustine 14% in part II with Sunitinib vs. 15% with Lomustine	6.5 months in part I with Sunitinib vs. 4.7 months with Lomustine 4.7 months in part II with Sunitinib vs. 7 months with Lomustine	Thrombocytopenia, fatigue, leukopenia, diarrhea, nausea, and vomiting
REGAL [288]	Cediranib alone/in combination with Lomustine vs. Lomustine plus placebo	III	325 patients with recurrent GBM	Cediranib alone (30 mg), Cediranib (20 mg) + Lomustine (110 mg/m^2^), or Lomustine (110 mg/m^2^) + placebo	16% Cediranib alone 35% Cediranib + Lomustine 25% Lomustine alone	8.0 months Cediranib alone, 9.4 months Cediranib + Lomustine 9.8 months Lomustine alone	Diarrhea, thrombocytopenia, neutropenia, and hypertension
De Groot, J.F [284]	Aflibercept	II	42 patients with GBM and 16 patients with anaplastic glioma	4 mg/kg on day 1 of every 2-week cycle.	7.7% for GBM	39 weeks	Fatigue, thromboembolia, wound healing, and CNS ischemia

## Data Availability

No new data were created or analyzed in this study. Data sharing is not applicable to this article.
